# Identification of concealed cardiomyopathy using next-generation sequencing–based genetic testing in Korean patients initially diagnosed with idiopathic ventricular fibrillation

**DOI:** 10.1093/europace/euad313

**Published:** 2023-11-10

**Authors:** Joo Hee Jeong, Yun Gi Kim, Suk-Kyu Oh, Hyoung Seok Lee, Yun Young Choi, Kyongjin Min, Jaemin Shim, Yae Min Park, Jun-Hyung Kim, Yong-Seog Oh, Nam-Ho Kim, Hui-Nam Pak, Young Keun On, Hyung Wook Park, Gyo-Seung Hwang, Dae-Kyeong Kim, Young-Ah Park, Hyoung-Seob Park, Yongkeun Cho, Seil Oh, Jong-Il Choi, Young-Hoon Kim

**Affiliations:** Division of Cardiology, Department of Internal Medicine, Korea University College of Medicine and Korea University Anam Hospital, 73, Goryeodae-ro, Seongbuk-gu, Seoul 02841, Republic of Korea; Division of Cardiology, Department of Internal Medicine, Korea University College of Medicine and Korea University Anam Hospital, 73, Goryeodae-ro, Seongbuk-gu, Seoul 02841, Republic of Korea; Division of Cardiology, Department of Internal Medicine, Korea University College of Medicine and Korea University Anam Hospital, 73, Goryeodae-ro, Seongbuk-gu, Seoul 02841, Republic of Korea; Division of Cardiology, Department of Internal Medicine, Korea University College of Medicine and Korea University Anam Hospital, 73, Goryeodae-ro, Seongbuk-gu, Seoul 02841, Republic of Korea; Division of Cardiology, Department of Internal Medicine, Korea University College of Medicine and Korea University Anam Hospital, 73, Goryeodae-ro, Seongbuk-gu, Seoul 02841, Republic of Korea; Division of Cardiology, Incheon Sejong General Hospital, Incheon, Korea; Division of Cardiology, Department of Internal Medicine, Korea University College of Medicine and Korea University Anam Hospital, 73, Goryeodae-ro, Seongbuk-gu, Seoul 02841, Republic of Korea; Department of Internal Medicine, Gachon University Gil Medical Center, Gachon University College of Medicine, Incheon, Korea; Department of Internal Medicine, Chungnam National University Hospital, Chungnam National University College of Medicine, Daejeon, Korea; Department of Internal Medicine, Seoul St.Mary’s Hospital, College of Medicine, The Catholic University of Korea, Seoul, Korea; Department of Internal Medicine, Wonkwang University Hospital, Wonkwang University School of Medicine, Iksan, Korea; Department of Internal Medicine, Severance Hospital, Yonsei University College of Medicine, Seoul, Korea; Department of Internal Medicine, Heart Vascular and Stroke Institute, Samsung Medical Center, Sungkyunkwan University School of Medicine, Seoul, Korea; Department of Cardiology, Chonnam National University Hospital, Chonnam National University School of Medicine, Gwangju, Korea; Department of Cardiology, Ajou University School of Medicine, Suwon, Korea; Department of Internal Medicine, Busan Paik Hospital, Inje University College of Medicine, Busan, Korea; Department of Internal Medicine, Busan Paik Hospital, Inje University College of Medicine, Busan, Korea; Department of Internal Medicine, Keimyung University Dongsan Medical Center, Keimyung University College of Medicine, Daegu, Korea; Department of Internal Medicine, Kyungpook National University Hospital, Daegu, Korea; Department of Internal Medicine, Seoul National University Hospital and Seoul National University College of Medicine, Seoul, Korea; Division of Cardiology, Department of Internal Medicine, Korea University College of Medicine and Korea University Anam Hospital, 73, Goryeodae-ro, Seongbuk-gu, Seoul 02841, Republic of Korea; Division of Cardiology, Department of Internal Medicine, Korea University College of Medicine and Korea University Anam Hospital, 73, Goryeodae-ro, Seongbuk-gu, Seoul 02841, Republic of Korea

**Keywords:** Idiopathic ventricular fibrillation, Genetic testing, High-throughput nucleotide sequencing, Channelopathy, Cardiomyopathy

## Abstract

**Aims:**

Idiopathic ventricular fibrillation (IVF) is a disease in which the cause of ventricular fibrillation cannot be identified despite comprehensive clinical evaluation. This study aimed to investigate the clinical yield and implications of genetic testing for IVF.

**Methods and results:**

This study was based on the multi-centre inherited arrhythmia syndrome registry in South Korea from 2014 to 2017. Next-generation sequencing–based genetic testing was performed that included 174 genes previously linked to cardiovascular disease. A total of 96 patients were clinically diagnosed with IVF. The mean age of the onset was 41.2 ± 12.7 years, and 79 patients were males (82.3%). Of these, 74 underwent genetic testing and four (5.4%) of the IVF probands had pathogenic or likely pathogenic variants (each having one of *MYBPC3*, *MYH7*, *DSP*, and *TNNI3*). All pathogenic or likely pathogenic variants were located in genes with definite evidence of a cardiomyopathy phenotype, either hypertrophic cardiomyopathy or arrhythmogenic right ventricular cardiomyopathy.

**Conclusion:**

Next-generation sequencing–based genetic testing identified pathogenic or likely pathogenic variants in 5.4% of patients initially diagnosed with IVF, suggesting that genetic testing with definite evidence genes of cardiomyopathy may enable molecular diagnosis in a minority of patients with IVF. Further clinical evaluation and follow-up of patients with IVF with positive genotypes are needed to unveil concealed phenotypes, such as the pre-clinical phase of cardiomyopathy.

## Graphical Abstract

**Graphical abstract:**
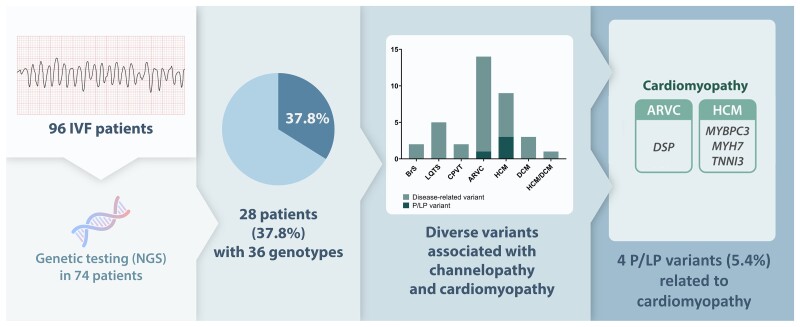
Concealed channelopathy and cardiomyopathy in IVF. IVF, idiopathic ventricular fibrillation; NGS, next-generation sequencing; P/LP, pathogenic or likely pathogenic; BrS, Brugada Syndrome; LQTS, long QT syndrome; CPVT, cathecholaminergic polymorphic ventricular tachycardia; ARVC, arrhythmogenic right ventricular cardiomyopathy; HCM, hypertrophic cardiomyopathy; DCM, dilated cardiomyopathy.

**What’s new?**

- In patients initially diagnosed with idiopathic ventricular fibrillation (IVF), next-generation sequencing–based genetic testing has identified 5.4% (4/74) of pathogenic or likely pathogenic variants that are associated with cardiomyopathy.
- Patients initially diagnosed with IVF may have concealed form of cardiomyopathy without evident phenotype.
- Further monitoring is needed to uncover the clinical phenotypes of cardiomyopathy in patients with IVF with cardiomyopathy-related genotypes.

## Introduction

Sudden cardiac arrest (SCA) is the unexpected and abrupt cessation of cardiac activity in a person without any prior fatal conditions. Sudden cardiac arrest is the leading cause of death worldwide, with ventricular fibrillation (VF) being a common electrocardiographic presentation.^1^ In addition to ischaemic heart disease, cardiomyopathies and inherited arrhythmia syndromes cause SCA, and an implantable cardioverter-defibrillator (ICD) is implanted for the secondary prevention of sudden cardiac death.^2,3^ Inherited arrhythmia syndromes are primary electrical disorders without an identifiable structural substrate, which are characterized by alterations in the genes that encode cardiac ion channel proteins. Inherited arrhythmia syndromes include Brugada syndrome (BrS), long QT syndrome (LQTS), short QT syndrome, early repolarization syndrome, catecholaminergic polymorphic ventricular tachycardia (CPVT), and idiopathic VF (IVF). Idiopathic ventricular fibrillation is a diagnosis of exclusion when the cause of VF cannot be identified despite a thorough clinical examination of structural, channelopathic, metabolic, or toxicologic aetiologies.^4^ The exact incidence of IVF is unclear but is expected to decrease with (i) the advances in contemporary imaging techniques [such as cardiac magnetic resonance (CMR) imaging] and (ii) current standardized recommendations for investigating unexplained SCA.^4,5^ Previously, most inherited arrhythmia syndromes, including BrS, short QT syndrome, and early repolarization syndrome, were regarded as IVF.^6^ Therefore, patients diagnosed with IVF are presumed to have an undiscovered or concealed form of inherited arrhythmia syndrome or cardiomyopathy with a genetic basis in channelopathy or cardiomyopathy.^7,8^

The underlying genotypes of patients diagnosed with IVF are diverse, and genetic testing is considered to identify causative genetic variants and uncover concealed causative diseases.^9^ It has been difficult to detect unknown disease-causing genotypes using traditional targeted genetic testing such as Sanger sequencing, owing to time and expense constraints. Next-generation sequencing (NGS) uses cyclic sequencing of massively parallel-aligned DNA fragments via clonal amplification,^10^ enabling it the screening of genetic variants that might be disease-causing and making it suitable for the genetic evaluation of patients with IVF. Hence, we comprehensively evaluated IVF probands using NGS to uncover concealed diseases and to further identify the clinical yield and implications of genetic testing in IVF.

## Methods

### Study subjects

This study was based on a multi-centre registry of inherited arrhythmia syndromes in South Korea. A total of 265 probands from 14 tertiary centres were enrolled between 2014 and 2017. Registry enrolment was performed using the diagnostic criteria in the 2013 Heart Rhythm Society (HRS)/European Heart Rhythm Association (EHRA)/Asia Pacific Heart Rhythm Society (APHRS) expert consensus statement.^11^ Genetic analysis by NGS was performed by obtaining a 20 mL blood sample from each enrolled patient. This study was approved by the Institutional Review Board of Korea University Anam Hospital (IRB No.2014AN0104), and written informed consent was obtained from all patients. The study protocol strictly adheres to the ethical guidelines of the Declaration of Helsinki and legal regulations of the Republic of Korea.

**Figure 1:**
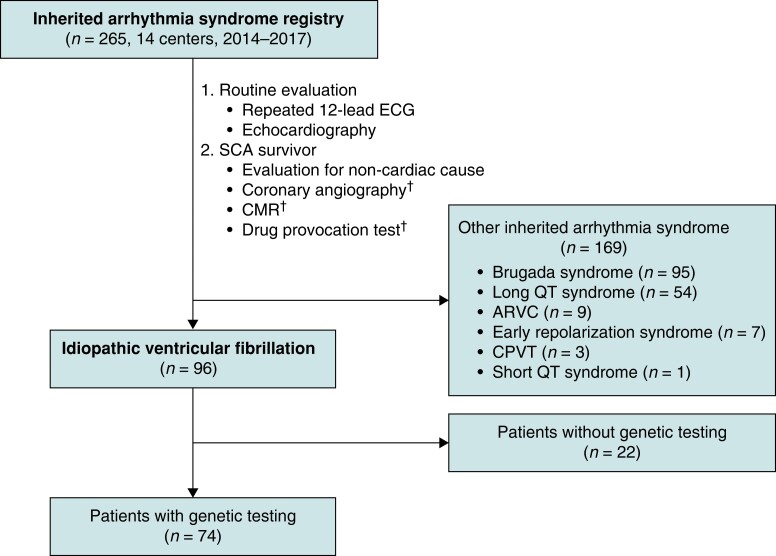
Flowchart of the study. †Coronary angiography was performed in all patients except those with little risk of coronary artery disease (such as under-aged or younger adults). CMR and drug provocation tests were conducted at the physician’s discretion in the local region according to the patients’ clinical circumstances and availability. ECG, electrocardiography; CMR, cardiac magnetic resonance imaging; VF, ventricular fibrillation.

IVF was defined as an unexplained, aborted SCA with shockable rhythms despite thorough clinical evaluation of underlying cardiac and non-cardiac aetiologies.^11^ Diagnosis of IVF was established after exclusion of possible cardiac causes for SCA: (i) obstructive coronary artery disease or vasospasm in patients at risk of coronary artery disease, (ii) evident heart failure or cardiomyopathy, and (iii) other overt inherited arrhythmia syndrome (including BrS, LQTS, or short QT syndrome). Patients with non-cardiac causes of SCA, such as hyperkalaemia or cerebral haemorrhage, were excluded from the study. Sudden cardiac arrest was evaluated based on current guidelines.^11^ After resuscitation from cardiac arrest, repeated 12-lead electrocardiography was performed, and laboratory testing and imaging work-up [such as chest X-ray, brain computed tomography (CT), or magnetic resonance imaging] were performed to exclude non-cardiac causes (*Figure 1*). Echocardiography and coronary (or CT) angiography were performed to rule out any relevant structural heart disease. Coronary (or CT) angiography was performed in all patients except those with little risk of coronary artery disease (such as under-aged or younger adults). Cardiac magnetic resonance imaging and drug provocation tests (including sodium channel blocker or epinephrine challenge test) were conducted at the physician’s discretion in the local region and according to the patients’ clinical circumstances and availability. Genetic testing was recommended for all patients, and those without informed consent for genetic analysis and their expenses were excluded from further genetic testing. If a certain genetic variant was detected, genetic counselling and screening of family members were performed. An ICD was implanted for secondary prevention of SCA.

Clinical data assessed at the time of resuscitation were obtained using an electronic case report form, which consisted of demographic data (height, body weight, date of diagnosis, medical records of hospitalizations, symptoms, history of ventricular arrhythmia, comorbidities, and family history), electrocardiography (one or more baseline 12-lead electrocardiographs), and echocardiography. Clinical data were reviewed by four cardiologists (J.H.J., Y.G.K., S.-K.O., and J.-I.C.) for their relevance to diagnosis, and results of genetic testing were analysed by three experts in bioinformatics (Green Cross Genome) and reappraised by four cardiologists.

### Next-generation sequencing–based genetic testing and bioinformatics

A multi-gene panel kit (3130 target lesions) manufactured by Illumina Design Studio (Illumina Inc., San Diego, CA, USA) was used to identify 174 genes detected in the Human Gene Mutation Database as causing cardiovascular disease (see Supplementary material online, *Table S1*). Pathogenicity was classified as pathogenic, likely pathogenic, uncertain significance, likely benign, or benign, based on the standard and guidelines for the interpretation of sequence variants of the American College of Medical Genetics and Genomics and the Association for Molecular Pathology.^12^ Further details about genetic testing and bioinformatics are provided in Supplementary material online, *Supplementary Methods*.

### Statistical analysis

The variables are described as numbers and percentages for categorical variables and as means ± standard deviation for continuous variables. Categorical and continuous variables were compared using the χ^2^ test, Fisher’s exact, Student’s *t*-test, or Mann–Whitney *U* tests, as indicated. All statistical analyses were performed using SPSS version 26 software (SPSS Inc., Chicago, IL, USA).

## Results

### Clinical characteristics

Among 265 probands with inherited arrhythmia syndrome or unexplained SCA, 96 were diagnosed with IVF (*Figure 1*). Seventy-four IVF probands were subjected to genetic testing using NGS to identify genotypes with unknown phenotypes. The baseline characteristics of the 96 patients with IVF and those who underwent genetic testing (*n* = 74) are described in *Table 1*. In IVF cohort (*n* = 96), the mean age of onset was 41.2 ± 12.7 years, and 82.3% of the patients were males. The mean QTc interval was 437.9 ± 43.9 ms, and the mean left ventricular ejection fraction was 56.9 ± 10.7 %. Majority of the patients received ICD implantation (88.5%). Eleven patients did not receive ICD implantation because of patient refusal or loss to follow-up (transferred to another hospital) before ICD implantation. Seven patients (7.3%) had family history of sudden cardiac death (<50 years). There were no significant differences in the baseline characteristics, demographics, corrected QTc interval, and left ventricular ejection fraction according to genetic testing result.

**Table 1:** Baseline characteristics

	Total (*n* = 96)	NGS (*n* = 74)	Gene positive^a^ (*n* = 28)	Gene negative (*n* = 46)
Age of onset (years)	41.2 ± 12.7	41.3 ± 13.4	45.1 ± 12.4	39.3 ± 13.6
Male sex	79 (82.3)	62 (83.8)	25 (89.3)	37 (80.4)
Hypertension	25 (26.0)	19 (25.7)	9 (32.1)	10 (21.7)
Diabetes mellitus	7 (7.3)	4 (5.4)	2 (7.1)	2 (4.3)
Chronic kidney disease	1 (1.0)	1 (1.4)	0 (0.0)	1 (2.2)
Dyslipidaemia	3 (3.1)	2 (2.7)	0 (0.0)	2 (4.3)
Ischaemic stroke	3 (3.1)	3 (4.1)	1 (3.6)	2 (4.3)
Haemorrhagic stroke	0 (0.0)	0 (0.0)	0 (0.0)	0 (0.0)
Family history of sudden cardiac death (<50 years)	7 (7.3)	7 (9.5)	3 (10.7)	4 (8.7)
Corrected QT interval (ms)	437.9 ± 43.9	438.9 ± 44.9	428.2 ± 41.4	447.4 ± 46.4
Left ventricular ejection fraction (%)	56.9 ± 10.7	55.4 ± 10.1	56.5 ± 10.4	54.6 ± 9.9
Coronary angiography or CT angiography	74 (77.1)	56 (75.7)	23 (82.1)	33 (71.7)
Cardiac magnetic resonance imaging	26 (27.1)	18 (23.7)	7 (25.0)	11 (23.9)
Sodium channel blocker challenge	15 (15.6)	13 (17.6)	5 (17.9)	8 (17.4)
Epinephrine challenge	33 (34.4)	19 (25.7)	7 (25.0)	12 (26.1)
ICD implantation	85 (88.5)	65 (87.8)	23 (82.1)	42 (91.3)

NGS, next-generation sequencing; CT, computed tomography; ICD, Implantable cardioverter-defibrillator.

^a^‘Genotype positive’ indicated rare variant carriers (pathogenic, likely pathogenic, or variants of unknown significance) that are related to channelopathy or cardiomyopathy.

### Genetic testing in idiopathic ventricular fibrillation

**Figure 2:**
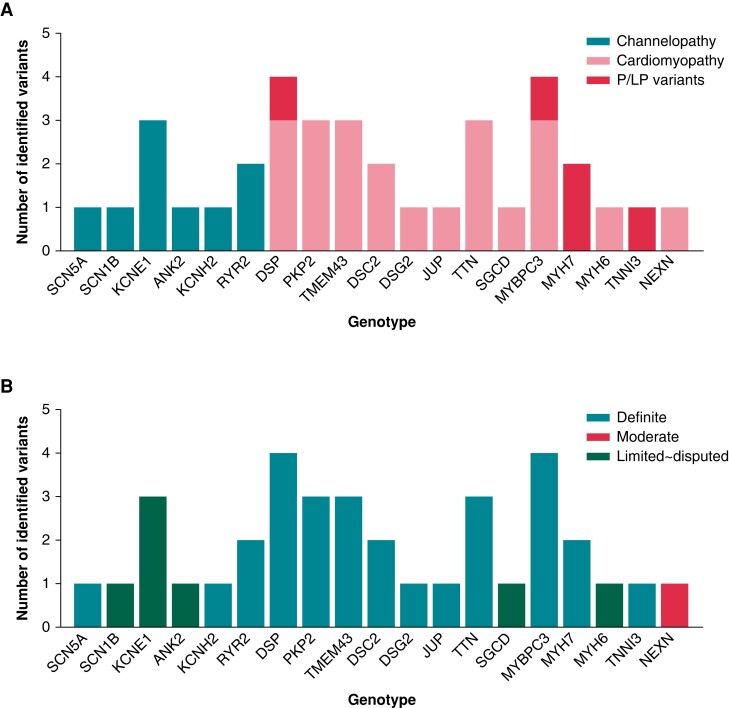
Results of next-generation sequencing–based genetic testing in idiopathic ventricular fibrillation probands. The identified variants were classified by channelopathy/cardiomyopathy and pathogenicity (P/LP) (*A*) and further categorized by the level of evidence supported by the gene/disease relationship curated by ClinGen (*B*). P/LP, pathogenic or likely pathogenic.

A schematic of the NGS test results is shown in *Figure 2*. Among the 74 patients with IVF who underwent genetic testing with an NGS panel, 28 (37.8%) were found to have genetic variants representing 36 variants, of which 9 (25.0%) were classified as channelopathy related and 27 (75.0%) were classified as cardiomyopathy related (*Figure 2A*). Four of the 36 identified variants were verified as pathogenic or likely pathogenic (P/LP) variants relevant to cardiomyopathy (*DSP, MYBPC3, MYH7, TNNI3*). The identified variants were further classified via gene disease validity based on the ClinGen Gene Curation Expert Panel (*Figure 2B*). Four of nine variants were located in genes classified as having definite evidence of channelopathy of BrS (*SCN5A*), LQTS (*KCNH2*), and CPVT (*RYR2*), whereas 24 of 27 variants were classified as definite evidence genes susceptible to arrhythmogenic right ventricular cardiomyopathy (ARVC; *DSP, PKP2, TMEM43, DSC2, DSG2, JUP*), hypertrophic cardiomyopathy (HCM; *MYBPC3, MYH7, TNNI3*), and dilated cardiomyopathy (DCM; *TTN*).^13–18^ One variant (*NEXN*) was moderate evidence gene related to DCM. Notably, all four P/LP cardiomyopathy-related variants were located in genes classified as providing definite evidence of cardiomyopathy according to the ClinGen Gene Curation Expert Panel.

### Channelopathy-related genetic variants

Nine patients harboured channelopathy-associated genetic variants (see Supplementary material online, *Table S2*). Two variants were classified as BrS associated, five as LQTS associated, and two as CPVT associated. None of the channelopathy-related variants were classified as P/LP variants. The channelopathy-related genotypes tended to have higher minor allele frequency in the Korean population database (KRGDB) than in the global population databases (GnomAD, ExAC, and 1000 Genome Project) (see Supplementary material online, *Table S3*). p.Asp85Asn located in *KCNE1* is a well-known polymorphism and was classified as of uncertain significance in this study because *in silico* analyses revealed conflicting results. However, previous studies have suggested that this genotype might express a mild phenotype of LQTS and exert loss-of-function effects on both *KCNQ1-*enhanced and *KCNH2-*encoded channel currents.^19^

### Cardiomyopathy-related genetic variants

Twenty-three patients had 27 cardiomyopathy-related genetic variants—14 ARVC related, 3 DCM related, and 9 HCM related variants (see Supplementary material online, *Table S4*). Four of these 27 variants (14.8%) were P/LP variants (*Table 2*). Although ARVC was the most commonly associated disease, accounting for 38.9% (14/36) of all identified genotypes, HCM showed the highest proportion of P/LP variants.

**Table 2:** Summary of pathogenic and likely pathogenic variants

No.	Sex	Age	QTc interval (ms)	LV EF (%)	Coronary angiography	CMR	Gene	Amino acid	Coding effect	Genetic diagnosis	Pathogenicity	Supporting ACMG criterion
1	M	25	419	57	Not done	LV hypokinaesia except basal lateral wall^a^	*DSP*	p.Ser711CysfsTer4	Splice acceptor	ARVC	Likely pathogenic	PVS1, PM2
2	M	75	484	52	No coronary stenosis	Not done	*MYBPC3*	p.Arg945GlyfsTer105	Frameshift	HCM	Pathogenic	PVS1, PM2, PP5
3	M	48	470	52	No coronary stenosis or vasospasm	Focal LGE at LV apex and lateral wall	*MYH7*	p.Arg719Gln	Missense	HCM	Pathogenic	PS4, PM1, PM2, PM5, PP1, PP2, PP3, PP5
4	M	38	384	63	No coronary stenosis or vasospasm	Normal	*TNNI3*	p.Arg145Gln	Missense	HCM	Pathogenic	PS3, PM1, PM5, PP2, PP3, PP5

LV EF, left ventricular ejection fraction; CMR, cardiac magnetic resonance imaging; ACMG, American College of Medical Genetics and Genomics; LGE, late gadolinium enhancement; ARVC, arrhythmogenic right ventricular cardiomyopathy; HCM, hypertrophic cardiomyopathy.

^a^This patient initially revealed severely reduced LV EF (22%) at the time of resuscitation, with CMR finding of regional LV wall hypokinaesia that is compatible with stress-induced cardiomyopathy. Follow-up echocardiography at the time of discharge revealed fully recovered LV EF (57%) without regional wall motion abnormalities.

Most ARVC-associated genotypes showed genetic alterations in the desmosomal genes (*DSC2, DSG2, DSP, PKP2,* and *JUP*). *DSP* was the most common gene (4/14, 28.6%), followed by *PKP2* (3/14, 21.4%) and *TMEM43* (3/14, 21.4%). One variant (*DSP* p.Ser711CysfsTer4) was classified as P/LP variant with absent or extremely low frequency in the population database (see Supplementary material online, *Table S5*). Most DCM-related variants belonged to the *titin* protein with missense variants, and none of the DCM-related variants were classified as P/LP variant. Among the HCM-related variants, three variants—one each of *MYBPC3* (p.Arg945GlyfsTer105), *MYH7* (p.Arg719Gln), and *TNNI3* (p.Arg145Gln)—were classified as P/LP variants. All, excluding one (*MYBPC3* p.Arg945GlyfsTer105), were missense variants. Notably, p.Arg719Gln in *MYH7* is a novel variant that lies in the head region of the protein and is absent from all population databases (GnomAD, ExaC, 1000 Genomes Project, KRGDB). It was classified as pathogenic by the ClinGen cardiomyopathy variant curation expert panel.^20^ All four patients with P/LP variant had preserved left ventricular ejection fraction, and three patients who underwent CMR revealed normal or minor abnormalities, regional wall motion abnormalities that did not fit coronary artery disease, or focal late gadolinium enhancement.

## Discussion

This study aimed to uncover the genetic predisposition in IVF probands and to identify clinical yield and implications of genetic testing in IVF. Genetic testing of patients initially diagnosed with IVF identified a non-trivial number of channelopathy-associated and cardiomyopathy-associated variants, i.e. variants in 28 patients (37.8%). Cardiomyopathy-related variants were detected more frequently than channelopathy-related variants (75.0% compared with 25.0%). However, only four cardiomyopathy-related variants were found to be P/LP.

Even in the absence of definite abnormalities in the underlying electrocardiography or echocardiography findings, genetic tests revealed channelopathy-related or cardiomyopathy-related genetic variants. These findings suggest that concealed channelopathy or cardiomyopathy can cause ventricular arrhythmia, even without clinical evidence of the disease.

### Channelopathy-related variants in idiopathic ventricular fibrillation

Most channelopathy-related variants are associated with BrS or LQTS. Other channelopathy-related variants such as CPVT, early repolarization syndrome, and short QT syndrome are relatively rare. This finding may be explained by the relatively high incidence of BrS and LQTS, especially BrS, in Asian populations, as well as the well-established genetic background of BrS and LQTS.^21^

Specific genotypes supported the clinical diagnosis of inherited arrhythmia syndrome. For instance, a pathogenic variant of *RYR2* or *CASQ2* is a diagnostic criterion that can independently confirm CPVT.^11^ In addition, an unequivocally pathogenic variant of LQTS supports the diagnosis of LQTS in patients with a corrected QT interval in the normal range.^11^ Therefore, the identification of specific genotypes could enable the molecular diagnosis of an inherited arrhythmia syndrome even without an apparent electrocardiographic abnormality at presentation.

### Concealed cardiomyopathy in idiopathic ventricular fibrillation

Among the cardiomyopathy-related variants, the most common relevant disease was ARVC; however, P/LP variants were most frequently detected among the HCM-related genotypes. Most ARVC-related variants are genes in the cardiac desmosome proteins that establish tight extracellular junctions between cells. The most common ARVC-related genotypes were in *DSP*, whereas *PKP2* is the most common genotype in the western population.^22^ Among the HCM-related variants, most genotypes involve variants in eight disease-causing genes encoding cardiac sarcomere proteins (*MYH7, MYBPC3, TNNI3, TPM1, MYL2, ACTC1, TNNT2,* and *MYL3*). In addition, most of the P/LP variants were missense variants, which are the major causal variant of HCM.^23^ Notably, one HCM-related genotype (p.Arg719Gln in *MYH7*) was identified as a disease-causing variant with a high level of evidence; therefore, its presence indicates a strong possibility of underlying HCM.^20^

Clinical ARVC is preceded by a pre-clinical phase that is characterized by minimal or no structural abnormalities, and SCA may be the first clinical presentation of this disease.^7,22^ Likewise, although HCM is the most common monogenic cardiovascular disease, it might not get diagnosed if the clinical diagnostic criteria are not fulfilled. In the present study, we identified a substantial proportion of cardiomyopathy-related variants, most of which were associated with ARVC and HCM. However, none of these patients presented with apparent cardiomyopathy. The CMR results in three patients with P/LP variants revealed only minor abnormalities that did not provide evidence of clinical cardiomyopathy. This finding supports the concept of a concealed or pre-clinical phase of cardiomyopathy, highlighting the importance of consistent monitoring for structural changes in these patients.^7^

### Next-generation sequencing–based genetic testing in idiopathic ventricular fibrillation

Idiopathic ventricular fibrillation is a diagnosis of exclusion in patient who follows a thorough evaluation of structural heart disease and inherited arrhythmia syndrome. Although recent genetic studies have revealed several causative genotypes in IVF (including *CALM1* and Dutch *DPP6*-haplotype), most patients with IVF do not have a clear genetic background.^24–26^ Previous studies revealed the genetic backgrounds of Caucasian patients with IVF, with multiple gene panels (NGS or whole exome sequencing) detecting 21–212 gene variants.^27–29^ However, the genetic yield of disease-causing variants in Caucasian patients with IVF was relatively low, ∼3–9%. Recent genetic study of European ancestry from cardiac arrest survivor with preserved ejection fraction registry identified ∼10% of P/LP variants via whole exome sequencing, in which majority of variants was found to be located in cardiomyopathy-associated genes.^29^ Furthermore, genetic testing of 24 strong evidence cardiomyopathy-susceptible genes in unexplained SCA survivors revealed higher proportion (18.4%) of relevant P/LP variants.^30^ Our study used genetic testing with a large volume of panel (174 genes) and revealed similar but relatively low yield of P/LP variants compared with previous studies, i.e. 5.4% of P/LP variants relevant to cardiomyopathy. In addition, all of the cardiomyopathy-related P/LP variants were definite evidence genes, as appraised by the ClinGen Gene Curation Expert Panel.

Genetic testing is recommended by current guidelines regarding patients with IVF, particularly in younger survivors (age < 50 years) or those in whom genetic causes are likely.^4,9^ Although genetic testing might provide supportive evidence in patients that lack the aetiology of SCA, use of a pan-arrhythmic panel or whole exome sequencing is unfavourable for clinical purpose because it increases the number of VUSs without a significant increase in P/LP variants and is not cost-effective. Variant of uncertain significance lacks additional diagnostic value that could support clinical decision-making, and an increase in VUSs increases the chances of secondary or incidental findings that are not relevant to the disease of interest.^31^ This may lead to unnecessary clinical investigations of the index proband, as well as unnecessary cascade screening of family members. More importantly, the notion of inherited cardiac disease and its uncertainty impose significant anxiety and psychological burden on the family for a long period until the gene–disease relationship is elucidated. Therefore, genetic testing of genes with strong to definite evidence of channelopathy or cardiomyopathy may be more suitable for clinical use. Genes with significant diagnostic value should also be considered, such as definite evidence genes for LQTS, CPVT, and ARVC.

Our findings support the clinical potential of NGS-based genetic testing for uncovering causative genetic variants in IVF, especially in genes with strong to definite evidence of susceptibility to cardiomyopathy. Furthermore, because patients with IVF express the most severe electrical phenotypes, consistent clinical investigations and genotype–phenotype correlation studies are needed in patients with genetic variants. An appropriate volume of genetic testing and proper interpretation of the genetic results are warranted in this population.

### Limitations

This study had several limitations. First, clinical data were obtained from a retrospective registry that assessed patients at the time of SCA, and a thorough evaluation to rule out possible causes of VF was not standardized among centres and different time periods. At the time of enrolment, there was no standardized approach to evaluate patients with unexplained SCA, which was similar to previous studies.^5^ For instance, not all patients were evaluated with coronary angiography. Unrecognized coronary anomalies or coronary vasospasm may predispose to transient ischaemia that leads to SCA.^32^ Similarly, the proportion of patients who underwent CMR imaging and drug provocation testing was relatively low. Among 23 patients with cardiomyopathy-related genotypes, only 7 were evaluated with CMR (see Supplementary material online, *Table S4*). Nonetheless, CMR in this population revealed non-specific findings related to stress-induced cardiomyopathy or minor abnormalities that were not relevant to clinical cardiomyopathies. The patients initially diagnosed with IVF may have included those with a pre-clinical phase of cardiomyopathy that was not evident during the initial phase. In this regard, a full evaluation at the initial phase of SCA and re-evaluation of the diagnosis with follow-up data might reveal concealed cardiomyopathy, which might be further indicative of each patient’s genetic background.

Secondly, the gene panel used in this study did not include recently established cardiomyopathy-associated gene such as filamin C (*FLNC*). Ortiz-Genga *et al.*^33^ reported that a truncal mutation of *FLNC* was identified as a causative gene of arrhythmogenic cardiomyopathy that involves the left ventricle or the phenotype of DCM. Recent studies have suggested its significance in SCA, and *FLNC* is currently included as a definitive evidence gene that should be screened for DCM probands.^9,14^ Evidence and recommendation for genetic testing change constantly, and designing gene panels that include key arrhythmogenic genes in a limited volume is challenging. Constant studies on genetic evidence for arrhythmogenic phenotypes may promote the development of cost-effective gene panels that accurately verify gene–disease relationship.

Thirdly, although we clinically investigated the family history of SCA or inherited arrhythmia syndrome in IVF probands, we did not conduct in-depth genetic investigations such as functional studies *in vitro* or in animal models, which might have weakened the reported pathogenicity of the genetic variants. In addition, for those with detected genetic variants, clinical surveillance of family members is recommended and only partially conducted; however, routine cascade genetic screening was not performed. A comprehensive interpretation of the clinical correlation among phenotypes, follow-up monitoring, and familial screening is required.

## Conclusions

Next-generation sequencing–based genetic testing can detect causal genetic variants up to 5.4% of patients initially diagnosed with IVF, suggesting that genetic testing with strong to definite evidence genes of cardiomyopathy may enable molecular diagnosis in patients with IVF. Genotypic identification can provide supporting evidence for therapeutic strategies and genetic counselling. Patients with positive genotypes should be monitored consistently to determine the underlying causes of their conditions.

## Supplementary Material

euad313_Supplementary_Data

## Data Availability

The data relevant to this study are available in the article.
