# Supplementary material for: Identification of concealed cardiomyopathy using next-generation sequencing–based genetic testing in Korean patients initially diagnosed with idiopathic ventricular fibrillation
Source: Europace. 2023 Nov 10;25(11):euad313. doi: 10.1093/europace/euad313 (PMC10639093; doi:10.1093/europace/euad313)
Supplement: euad313_Supplementary_Data [file euad313_supplementary_data.docx]

**SUPPLEMENTARY MATERIAL**

**Supplementary Methods. NGS-based Genetic Testing and Bioinformatics**

**Supplementary Table 1. Next-generation sequencing panel (Illumina, Miseq) with 174 cardiovascular disease–causing genetic variants**

**Supplementary Table 2. Clinical characteristics and genetic testing results in channelopathy-related genotypes**

**Supplementary Table 3. Genetic analysis of channelopathy-related genotypes**

**Supplementary Table 4. Clinical characteristics and genetic testing results in cardiomyopathy-related genotypes**

**Supplementary Table 5. Genetic analysis of cardiomyopathy-related genotypes**

**Supplementary Methods. NGS-based Genetic Testing and Bioinformatics**

Genomic DNA was extracted from peripheral leukocytes using a Wizard Genomic DNA Purification kit (Promega, Madison, WI, USA). A Celemics Customized Target Enrichment kit (Celemics Inc, Seoul, Korea) was used for library preparation. The Illumina MiSeq platform (Illumina Inc., San Diego, CA, USA) was used to generate 2 x 150-bp paired-end reads. The alignment of sequence reads, indexing of the reference genome, hg19 (GRCh37), and variant calling were conducted with a pipeline based on the Genome Analysis Tool Kit (GATK) Best Practice. Alignment was done with BWA-mem (version 0.7.12); duplicated reads were marked with Picard (version 1.96, http://picard.sourceforge.net); local alignment, base quality recalibration, and variant calling were performed with GATK, (version 3.2-2); and annotation was done with VEP (Variant Effect Predictor), dbNSFP v3.02.

Data were analyzed using the following three steps. The primary analysis was sequence generation, which converted the sequenced raw data to a text-based format (FASTQ) suitable for storing both biological sequences and the corresponding quality scores. The secondary analysis was sequence processing with variant calling, which selected sequences (variants) that differed from the reference sequences. The tertiary analysis was validation and clinical interpretation in light of databases and algorithms (evidence based, frequency-based, functional, and predictive).

**Bioinformatics**

Filtering of false-positive variants was performed as follows. The region coding the actual protein was distinguished from the flanking region, and only loss-of-function variants (frameshift, nonsense, missense, consensus splice site) were selected to distinguish the mutations causing the actual variant. Variant allele frequencies of more than 0.3 were included. Compound heterozygous variants in autosomal recessive inheritance genes were included, as were disease causing variants in HGMD. Analysis accuracy was validated as follows. Each read depth was 10X or higher to ensure sensitivity; only variants with 10X coverage of 90% or more were included; and the number of calling variants was at least 100 per batch.

Multiple databases were used to analyze the variants. Minor allele frequency was determined according to the following population databases: Genome Aggregation Database (gnomAD, <https://gnomad.broadinstitute.org/>), 1000 Genome Project (http://www.internationalgenome.org/), the Exome Aggregation Consortium (ExAC, http://exac.broadinstitute.org), and Korean reference genome database (KRGDB, http://coda.nih.go.kr/coda/KRGDB/index.jsp). Minor allele frequencies of more than 0.01 in any population frequency databases (gnomAD, ExAC, 1000 genome project, KRGDB) were excluded.

The association with inherited arrhythmia or cardiac disease was determined using disease databases: HGMD, Clinvar (https://www.ncbi.nlm.nih.gov/clinvar/), and OMIM (http://www.omim.org/). Eight different *in silico* analysis tools were used to predict pathogenicity of missense variant: SIFT (https://sift.bii.a-star.edu.sg/), Polyphen2 (HDIV/HVAR) (http://genetics.bwh.harvard.edu/pph2/), likelihood-ratio test, Mutation Taster/Assessor (http://www.mutationtaster.org/, http://mutationassessor.org/), FATHMM (http:// fathmm.biocompute.org.uk), and PROVEAN (http:// provean.jcvi.org).

**Supplementary Table 1. Next-generation sequencing panel (Illumina, Miseq) with 174 cardiovascular disease–causing genetic variants**

| **Disease group** | **Disease** | **Gene** |
| --- | --- | --- |
| Inherited arrhythmia | Long QT syndrome | *AKAP9, ANK2, CACNA1C, CALM1, CAV3, KCNE1, KCNE2, KCNE3, KCNH2, KCNJ2, KCNJ5, KCNQ1, RYR2, SCN4B, SCN5A, SNTA1* |
|  | Short QT syndrome | *CACNA2D1, KCNH2, KCNJ2, KCNQ1* |
|  | Brugada syndrome | *ABCC9, CACNA1C, CACNA2D1, CACNB2, GPD1L, HCN4, KCND3, KCNE3, KCNH2, KCNJ8, PKP2, RANGRF, SCN2B, SCN3B, SCN5A, TRPM4* |
|  | Catecholaminergic polymorphic ventricular tachycardia | *CALM2, CASO2, KCNE1, KCNJ2, RYR2, TRDN* |
| Cardiomyopathy | Hypertrophic cardiomyopathy | *ACTA1, ACTC1, ACTN2, ANKRD1, BRAF, CACNA1C, CALR3, CASQ2, CAV3, COX15, CRYAB, CSRP3, DES, FHL1, FHL2, FXN, GAA, GLA, HRAS, JPH2, KCNQ1, KLF10, LAMP2, MAP2K1, MAP2K2, MYBPC3, MYH6, MYH7, MYL2, MYL3, MYLK2, MYO6, MYOZ2, MYPN, NEXN, PDLIM3, PRKAG2, PTPN11, RAF1, SLC25A4, SOS1, TCAP, TNNC1, TNNI3, TNNT2, TPM1, TRIM63, TTN, VCL* |
|  | Dilated cardiomyopathy | *ABCC9, ACTA1, ACTC1, ACTN2, ANKRD1, BAG3, CRYAB, CSRP3, CTF1, DES, DMD, DNAJC19, DOLK, DSC2, DSG2, DSP, EMD, EYA4, FKRP, FKTN, GATAD1, ILK, JUP, LAMA2, LAMA4, LAMP2, LDB3, LMNA, MURC, MYBPC3, MYH6, MYH7, MYPN, NEXN, NPPA, PDLIM3, PKP2, PLN, PRDM16, RBM20, SCN5A, SGCB, SGCD, TAZ, TCAP, TMPO, TNNC1, TNNI3, TNNT2, TPM1, TTN, TXNRD2, VCL, ZBTB17* |
|  | Arrhythmogenic right ventricular cardiomyopathy | *DES, DSC2, DSG2, DSP, JUP, LMNA, PKP2, RYR2, SCN5A, RGFB3, TMEM43, TTN* |
|  | Restrictive cardiomyopathy | *ACTC1, DES, HFE, MYH7, MYL2, MYL3, MTPN, TNNI3, TNNT2, TPM1,* |
|  | Left ventricular non-compaction | *ACTC1, CASQ2, DTNA, MIB1, MYBPC3, MYH7, PRDM16, TAZ, TNNT2, TPM1* |
|  | Noonan syndrome | *BRAF, CBL, KRAS, MAP2K1, NRAS, PTPN11, RAF1, SHOC2, SOS1* |
| Aortopathy | Marfan syndrome | *CBS, FBN1, LTBP2, TGFBR1, TGFBR2* |
|  | Loeys-Dietz syndrome | *FBN1, TGFBR1, TGFBR2* |
|  | Familial aortic aneurysm | *ACTA2, COL3A1, EFEMP2, FBN1, MYH11, MYLK, NOTCH1, SLC2A10, SMAD3, TGFB2, TGFBR1, TGFBR2* |
|  | Aortic valve disease | *ELN, FBN1, NOTCH1* |
| Other cardiac disease | Familial hypercholesterolemia | *ABCG5, ABCG8, APOA4, APOA5, APOB, APOC2, APOE, CETP, CREB3L3, GCKR, GPIHBP1, LDLR, LDLRAP1, LMF1, LPL, PCSK9, SREBF2, ZHX3* |
|  | Other | *ALMS1, COL5A1, COL5A2, CRELD1, DPP6, FBN2, GJA5, HADHA, HSPB8, JAG1, KCNA5, NKX2-5, NODAL, PRKAR1A, RYR1, SALL4, SCN1B, SCO2, SDHA, SEPN1, SMAD4, TBX3, TBX5, TBX20, TTR, ZIC3* |

**Supplementary Table2. Clinical characteristics and genetic testing results in channelopathy-related genotypes**

| **No.** | **Sex** | **Age of onset** | **Family history of SCA** | **Electrocardiography** | | **Echocardiography** | | | **Gene** | **Mutation** | **Amino acid** | **Nucleotide change** | **Coding effect** |
| --- | --- | --- | --- | --- | --- | --- | --- | --- | --- | --- | --- | --- | --- |
|  |  |  |  | **QTc interval (ms)** | **Other abnormality** | **LVEDD (mm)** | **LVESD (mm)** | **LV EF (%)** |  |  |  |  |  |
| 1^a^ | M | 75 | No | 484 | None | 52 | 34 | 52 | *KCNH2* | chr7-150644455-A-ACGTCGC | p.Gly1036_Asp1037dup | c.3107_3112dup | Duplication |
| 2 | M | 45 | No | 479 | None | 55 | 35 | 66 | *ANK2* | chr4-114284595-T-A | p.Trp3620Arg | c.10858T>A | Missense |
| 3 | M | 48 | No | 404 | None | 52 | 35 | 60 | *SCN1B* | chr19-35530138-C-T | p.Thr189Met | c.566C>T | Missense |
| 4^a^ | M | 38 | No | 384 | None | 53 | 32 | 63 | *SCN5A* | chr3-38647627-C-T | p.Ala385Thr | c.1153G>A | Missense |
| 5 | M | 42 | Yes | 452 | Epsilon wave | 50 | 34 | 62 | *KCNE1* | chr21-35821680-C-T | p.Asp85Asn | c.253G>A | Missense |
| 6 | M | 49 | Yes | NA | None | NA | NA | 66 | *KCNE1* | chr21-35821680-C-T | p.Asp85Asn | c.253G>A | Missense |
| 7^a^ | M | 51 | No | NA | None | NA | NA | NA | *KCNE1* | chr21-35821680-C-T | p.Asp85Asn | c.253G>A | Missense |
| 8 | F | 38 | No | 467 | None | 49 | 34 | 52 | *RYR2* | chr1-237729972-C-T | p.Thr1107Met | c.3320C>T | Missense |
| 9^a^ | M | 33 | No | 466 | Epsilon wave | 50 | 32 | 61 | *RYR2* | chr1-237777856-G-C | p.Val1810Leu | c.5428G>C | Missense |

^a^Four patients were detected with concurrent cardiomyopathy-related genotypes.

M, male; F, female; SCA, Sudden cardiac arrest; LVEDD, Left ventricular end-diastolic dimension; LVESD, Left ventricular end-systolic dimension; LV EF, Left ventricular ejection fraction; NA, Data not available

**Supplementary Table 3. Genetic analysis of channelopathy-related genotypes**

| **No.** | **dbSNP147** | **Genetic Diagnosis** | **Pathogenicity** | **Variant allele frequency** | **ExAC** | **GnomAD** | **GnomAD (East Asian)** | **1000 Genome Project** | **KRGDB** | **SIFT** | **PolyPhen HVAR** | **Mutation Taster** | **PROVEAN** | **PolyPhen HDIV** | **LRT** | **Mutation Assessor** | **FATHMM** |
| --- | --- | --- | --- | --- | --- | --- | --- | --- | --- | --- | --- | --- | --- | --- | --- | --- | --- |
| 1^a^ | rs1554424038 | LQTS | Uncertain significance | 0.40 | 0.00000 | 0.00001 | . | 0.00000 | 0.00045 | D | . | D | D | . | D | H | D |
| 2 | rs199473346 | LQTS | Uncertain significance | 0.39 | 0.00003 | 0.00003 | 0.00038 | 0.00000 | 0.00088 | D | D | D | D | . | D | H | D |
| 3 | rs2305748 | BrS | Uncertain significance | 0.59 | 0.00017 | 0.00019 | 0.00185 | 0.00060 | 0.00379 | D | P | B | N | D | D | L | D |
| 4^a^ | rs771588294 | BrS | Uncertain significance | 0.47 | 0.00001 | 0.00002 | 0.00005 | 0.00000 | 0.00029 | D | D | D | D | . | D | N | D |
| 5 | rs1805128 | LQTS | Uncertain significance | 0.45 | 0.00920 | 0.00932 | 0.00557 | 0.00379 | 0.00932 | D | B | B | D | P | N | M | D |
| 6 | rs1805128 | LQTS | Uncertain significance | 0.50 | 0.00920 | 0.00932 | 0.00557 | 0.00379 | 0.00932 | D | B | B | D | P | N | M | D |
| 7^a^ | rs1805128 | LQTS | Uncertain significance | 0.51 | 0.00920 | 0.00932 | 0.00557 | 0.00379 | 0.00932 | D | B | B | D | P | N | M | D |
| 8 | rs200236750 | CPVT | Uncertain significance | 0.43 | 0.00030 | 0.00043 | 0.00010 | 0.00000 | 0.00000 | D | B | D | N | P | N | N | T |
| 9^a^ | rs754364233 | CPVT | Uncertain significance | 0.53 | 0.00008 | 0.00008 | 0.00111 | 0.00000 | 0.00204 | D | B | D | N | P | N | M | T |

^a^Four patients were detected with concurrent cardiomyopathy-related genotypes.

BrS, Brugada syndrome; LQTS, Long QT syndrome; CPVT, Catecholaminergic polymorphic ventricular tachycardia.

SIFT, FATHMM: D, Deleterious; T, Tolerated,

PolyPhen: D, Probably damaging; P, Possibly damaging; B, Benign; U, Unknown,

LRT: D, Deleterious; N, Neutral; U, Unknown,

Mutation Taster: D, Disease causing; B, Benign (Polymorphism),

Mutation Assessor: N, Neutral; L, Low; M, Medium; H, High,

PROVEAN: D, Deleterious; N, Neutral.

**Supplementary Table 4. Clinical characteristics and genetic testing results in cardiomyopathy-related genotypes**

| **No.** | **Sex** | **Age of onset** | **Family history of SCA** | **Electrocardiography** | | **Echocardiography** | | | **Gene** | **Mutation** | **Amino acid** | **Nucleotide change** | **Coding effect** | **CMR** |
| --- | --- | --- | --- | --- | --- | --- | --- | --- | --- | --- | --- | --- | --- | --- |
|  |  |  |  | **QTc interval (ms)** | **Other abnormality** | **LVEDD (mm)** | **LVESD (mm)** | **LV EF (%)** |  |  |  |  |  |  |
| 1 | M | 47 | No | 445 | None | NA | NA | NA | *DSC2* | chr18-28648997-GTCC-G | p.Gly790del | c.2365GGA[1] | Inframe deletion |  |
| 2 | M | 22 | No | 443 | None | 50 | 35 | 59 | *DSC2* | chr18-28648997-GTCC-G | p.Gly790del | c.2365GGA[1] | Inframe deletion | Normal |
| 3 | M | 25 | NA | 419 | None | 44 | 34 | 57 | *DSP* | chr6-7574313-TGACA-T | p.Ser711CysfsTer4 | c.2131-3_2131delCAGA | Splice acceptor | LV hypokinesia except basal lateral wall (compatible to stress induced cardiomyopathy) |
| 4^a^ | M | 75 | No | 484 | None | 52 | 34 | 52 | *MYBPC3* | chr11-47356663-CCG-C | p.Arg945GlyfsTer105 | c.2833_2834del | Frameshift |  |
| 5 | M | 17 | Yes | 339 | None | 41 | 24 | 58 | *MYBPC3* | chr11-47367848-C-T | p.Glu334Lys | c.1000G>A | Missense | Focal fibrosis at LV wall |
| 6 | M | 48 | No | 470 | None | 45 | 30 | 52 | *MYH7* | chr14-23895179-C-T | p.Arg719Gln | c.2156G>A | Missense | Focal LGE at LV apex and lateral wall |
| 7 ^a^ | M | 38 | No | 384 | None | 53 | 32 | 63 | *TNNI3* | chr19-55665513-C-T | p.Arg145Gln | c.434G>A | Missense | Normal |
| 8 | M | 55 | NA | 414 | None | 46 | 28 | 68 | *JUP* | chr17-39925711-C-T | p.Ala143Thr | c.427G>A | Missense |  |
| 9 | M | 44 | No | 385 | Type 2 Brugada ECG | 44 | 26 | 58 | *DSG2* | chr18-29116222-A-C | p.Asp494Ala | c.1481A>C | Missense |  |
|  |  |  |  |  |  |  |  |  | *DSP* | chr6-7556049-A-G | p.Gln90Arg | c.269A>G | Missense |  |
| 10 | M | 48 | No | 389 | None | 44 | 31 | 55 | *MYBPC3* | chr11-47354443-G-A | p.Arg1138Cys | c.3412C>T | Missense |  |
| 11 | M | 24 | No | 450 | None | 41 | 29 | 58 | *MYH7* | chr14-23884227-G-A | p.Arg1846Cys | c.5536C>T | Missense |  |
| 12 | M | 50 | No | 418 | None | 69 | 66 | 20 | *NEXN* | chr1-78392548-C-T | p.Arg279Cys | c.835C>T | Missense | LGE at LV lateral wall |
|  |  |  |  |  |  |  |  |  | *MYBPC3* | chr11-47359040-C-A | p.Arg835Leu | c.2504G>T | Missense |  |
| 13 ^a^ | M | 33 | No | 466 | Epsilon wave | 50 | 32 | 61 | *DSP* | chr6-7580466-T-G | p.Leu1348Arg | c.4043T>G | Missense |  |
| 14 | M | 43 | Yes | 445 | Epsilon wave | 55 | 37 | 61 | *PKP2* | chr12-32955486-G-A | p.Pro717Leu | c.2150C>T | Missense |  |
| 15 | M | 66 | No | 459 | None | 42 | 29 | 40 | *PKP2* | chr12-32955486-G-A | p.Pro717Leu | c.2150C>T | Missense |  |
| 16 | F | 45 | No | 427 | None | 49 | 38 | 52 | *TMEM43* | chr3-14172430-A-G | p.Ile91Val | c.271A>G | Missense |  |
| 17 | F | 58 | No | 332 | None | 50 | 38 | 41 | *TMEM43* | chr3-14180693-G-C | p.Arg299Thr | c.896G>C | Missense |  |
|  |  |  |  |  |  |  |  |  | *TMEM43* | chr3-14172424-G-A | p.Val89Met | c.265G>A | Missense |  |
| 18 ^a^ | M | 51 | No | NA | None | NA | NA | NA | *MYH6* | chr14-23862177-C-G | p.Gln1065His | c.3195G>C | Missense |  |
| 19 | M | 34 | No | 392 | None | 49 | 25 | 60 | *TTN* | chr2-179602841-C-T | p.Ser4609Asn | c.14339G>A | Missense | Normal |
| 20 | M | 19 | No | 413 | None | 47 | 29 | 63 | *TTN* | chr2-179666973-C-T | p.Ala63Thr | c.187G>A | Missense |  |
| 21 | M | 53 | No | 495 | None | 53 | 33 | 65 | *TTN* | chr2-179667000-C-T | p.Val54Met | c.160G>A | Missense |  |
|  |  |  |  |  |  |  |  |  | *SGCD* | chr5-156186376-A-G | p.Gln282Arg | c.848A>G | Missense |  |
| 22 | M | 47 | No | 427 | None | 53 | 36 | 67 | *DSP* | chr6-7585950-A-C | p.Met2819Leu | c.8455A>C | Missense |  |
| 23 | M | 52 | No | 436 | None | 56 | 38 | 58 | *PKP2* | chr12-33031214-C-T | p.Val200= | c.600G>A | Synonymous |  |

^a^Four patients were detected with concurrent channelopathy-related genotypes.

M, male; F, female; SCA, Sudden cardiac arrest; LVEDD, Left ventricular end-diastolic dimension; LVESD, Left ventricular end-systolic dimension; LV EF, Left ventricular ejection fraction; CMR, cardiac magnetic resonance imaging; NA, Data not available; LV, left ventricle; LGE, late gadolinium enhancement.

**Supplementary Table 5. Genetic analysis of cardiomyopathy-related genotypes**

| **No.** | **dbSNP147** | **Genetic diagnosis** | **Pathogenicity** | **Variant allele frequency** | **ExAC** | **GnomAD** | **GnomAD (East Asian)** | **1000 Genome Project** | **KRGDB** | **SIFT** | **PolyPhen HVAR** | **Mutation Taster** | **PROVEAN** | **PolyPhen HDIV** | **LRT** | **Mutation Assessor** | **FATHMM** |
| --- | --- | --- | --- | --- | --- | --- | --- | --- | --- | --- | --- | --- | --- | --- | --- | --- | --- |
| 1 | rs377272752 | ARVC | Benign | 0.47 | 0.00000 | 0.00137 | 0.01791 | 0.00000 | 0.00000 | . | . | . | . | . | . | . | . |
| 2 | rs377272752 | ARVC | Benign | 0.46 | 0.00000 | 0.00137 | 0.01791 | 0.00000 | 0.00000 | . | . | . | . | . | . | . | . |
| 3 | rs746177210 | ARVC | Likely Pathogenic | 0.47 | 0.00001 | 0.00000 | 0.00000 | 0.00000 | 0.00000 | . | . | . | . | . | . | . | . |
| 4^a^ | rs397515987 | HCM | Pathogenic | 0.51 | 0.00000 | 0.00000 | 0.00006 ^b^ | 0.00000 | 0.00000 | . | . | . | . | . | . | . | . |
| 5 | rs573916965 | HCM | Uncertain significance | 0.51 | 0.00030 | 0.00009 | 0.00334 | 0.00100 | 0.00553 | D | B | D | D | P | . | M | T |
| 6 | rs121913641 | HCM | Pathogenic | 0.55 | 0.00000 | 0.00000 | . | 0.00000 | 0.00000 | D | B | D | N | P | . | M | D |
| 7^a^ | rs397516349 | HCM | Pathogenic | 0.46 | 0.00002 | 0.00002 | 0.00011 | 0.00000 | 0.00059 | D | D | D | D |  | D | M | D |
| 8 | rs375788626 | ARVC | Uncertain significance | 0.49 | 0.00011 | 0.00008 | 0.00015 | 0.00000 | 0.00000 | D |  | D | D | T | D | M | T |
| 9 | rs193298428 | ARVC | Uncertain significance | 0.55 | 0.00000 | 0.00003 | 0.00039 | 0.00000 | 0.00088 | D | D | D | D | D | D | H | T |
|  | rs188516326 | ARVC | Uncertain significance | 0.56 | 0.00068 | 0.00067 | 0.00898 | 0.00100 | 0.00553 | D | P | D | N | P | D | N | T |
| 10 | rs377171707 | HCM | Uncertain significance | 0.5 | 0.00004 | 0.00004 | 0.00018 | 0.00000 | 0.00029 | D | D | D | D | . | . | H | D |
| 11 | rs12590294 | HCM | Uncertain significance | 0.4 | 0.00002 | 0.00001 | 0.00005 | 0.00000 | 0.00000 | T | D | D | D | D | . | H | D |
| 12 | rs146245480 | HCM | Uncertain significance | 0.45 | 0.00000 | 0.00050 | 0.00630 | 0.00120 | 0.00524 | D | D | D | N | . | D | M | T |
|  | rs527305885 | HCM | Uncertain significance | 0.43 | 0.00000 | 0.00005 | 0.00072 | 0.00040 | 0.00088 | D | P | D | D | . | . | M | T |
| 13^a^ | rs767003564 | ARVC | Uncertain significance | 0.53 | 0.00002 | 0.00002 | 0.00016 | 0.00000 | 0.00233 | D | D | D | D | D | D | L | D |
| 14 | rs144018320 | ARVC | Uncertain significance | 0.51 | 0.00010 | 0.00012 | 0.00130 | 0.00060 | 0.00582 | D | D | D | D | D | N | M | T |
| 15 | rs144018320 | ARVC | Uncertain significance | 0.46 | 0.00010 | 0.00012 | 0.00130 | 0.00060 | 0.00582 | D | D | D | D | D | N | M | T |
| 16 | rs144811578 | ARVC | Uncertain significance | 0.35 | 0.00006 | 0.00008 | 0.00082 | 0.00020 | 0.00553 | T | B | B | N | B | N | N | T |
| 17 | rs139590716 | ARVC | Uncertain significance | 0.5 | 0.00050 | 0.00045 | 0.00501 | 0.00120 | 0.00671 | T | B | D | N | P | D | L | T |
|  | rs147319971 | ARVC | Uncertain significance | 0.49 | 0.00009 | 0.00009 | 0.00103 | 0.00040 | 0.00233 | D | P | D | D | D | D | M | T |
| 18^a^ | rs267606904 | HCM | Uncertain significance | 0.45 | 0.00023 | 0.00031 | 0.00167 | 0.00000 | 0.00262 | D | D | D | D | D | . | M | D |
| 19 | rs147879266 | DCM | Uncertain significance | 0.45 | 0.00013 | 0.00009 | 0.00103 | 0.00020 | 0.00233 | T | P | B | N | P | . | . | T |
| 20 | rs764892312 | DCM | Uncertain significance | 0.45 | 0.00004 | 0.00004 | 0.00035 | 0.00000 | 0.00320 | T | D | D | N | D | . | . | T |
| 21 | rs139517732 | DCM | Uncertain significance | 0.46 | 0.00005 | 0.00004 | 0.00055 | 0.00060 | 0.00175 | T | B | B | N | P | . | . | T |
|  | rs397516338 | HCM/DCM | Uncertain significance | 0.57 | 0.00047 | 0.00054 | 0.00758 | 0.00000 | 0.00786 | T | D | D | N | D | D | M | D |
| 22 | rs138329459 | ARVC | Likely Benign | 0.56 | 0.00172 | 0.00190 | 0.00241 | 0.00180 | 0.00233 | T | B | B | N |  | N | N | T |
| 23 | rs781305034 | ARVC | Likely Benign | 0.56 | 0.00009 | 0.00007 | 0.00105 | 0.00000 | 0.00524 | . | . |  | . | . | . | . | . |

^a^Four patients were detected with concurrent channelopathy-related genotypes.

^b^1 allele was found in 17,786 alleles of East Asian population.

ARVC, arrhythmogenic right ventricular cardiomyopathy; HCM, hypertrophic cardiomyopathy; DCM, dilated cardiomyopathy.

SIFT, FATHMM: D, Deleterious; T, Tolerated,

PolyPhen: D, Probably damaging; P, Possibly damaging; B, Benign; U, Unknown,

LRT: D, Deleterious; N, Neutral; U, Unknown,

Mutation Taster: D, Disease causing; B, Benign (Polymorphism),

Mutation Assessor: N, Neutral; L, Low; M, Medium; H, High,

PROVEAN: D, Deleterious; N, Neutral.
